# p‐Type TiO_2_ Nanotubes: Quantum Confinement and Pt Single Atom Decoration Enable High Selectivity Photocatalytic Nitrate Reduction to Ammonia

**DOI:** 10.1002/anie.202415865

**Published:** 2025-03-30

**Authors:** Hayoon Jung, Hyesung Kim, Johannes Will, Erdmann Spiecker, Patrik Schmuki

**Affiliations:** ^1^ Department of Materials Science and Engineering, WW4‐LKO Friedrich‐Alexander‐Universität Erlangen‐Nürnberg Martensstraße 7 91058 Erlangen Germany; ^2^ Regional Centre of Advanced Technologies and Materials Palacký University Šlechtitelů 27 Olomouc 78371 Czech Republic; ^3^ Institute of Micro‐ and Nanostructure Research & Center for Nanoanalysis and Electron Microscopy (CENEM), IZNF Friedrich‐Alexander‐Universität Erlangen‐Nürnberg Cauerstraße 3 91058 Erlangen Germany

**Keywords:** Photocatalytic nitrate reduction, Pt single atoms, p‐type titanium dioxide, Quantum confinement

## Abstract

We synthesize p‐type TiO_2_ nanotubes that allow band‐gap adjustment by quantum confinement. These tubes therefore enable reductive photocatalytic reactions that are not thermodynamically possible on classic titania photocatalysts. Here, we demonstrate the direct photocatalytic nitrate reduction to ammonia without any need of hole scavengers. The quantum confinement effect (and thus the thermodynamic driving force) can be controlled by the thickness of the nanotube walls. Notably, the use of Pt single atoms as cocatalysts decorated on the TiO_2_ nanotubes additionally offers a superior ammonia production and a remarkable enhanced selectivity compared to Pt nanoparticles. Overall, the work not only highlights the potential of size‐controlled modifications of electronic properties in extending the utility of a most classical photocatalyst but also exemplifies its use in technologically relevant reactions.

## Graphical abstract

p‐type TiO_2_ nanotubes with quantum confinement effect enable photocatalytic nitrate reduction to ammonia. The decoration with Pt single atoms significantly enhances the photocatalytic activity and selectivity toward ammonia while the quantum confinement effect and p‐type conductivity of the nanotubes maintain their key roles in the photocatalytic performance.

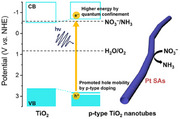

Titanium dioxide (TiO_2_) is the benchmark photocatalyst for its unparalleled chemical stability, prominent photocatalytic capabilities, and inherent nontoxicity.^[^ ^1^, ^2^, ^3^, ^4^ ^]^ TiO_2_ is intrinsically an n‐type semiconductor with a band gap from 3.0 to 3.2 eV, facilitating UV‐driven photocatalysis. It is widely used in essential reactions in environmental and energy sectors, such as pollutant degradation, water splitting, and CO_2_ reduction.^[^ ^5^, ^6^, ^7^, ^8^ ^]^ To enhance the photocatalytic efficiency of titania, extensive research has explored the avenues of nanostructuring and electronic modifications, aiming to optimize charge carrier dynamics.^[^ ^9^, ^10^, ^11^, ^12^ ^]^

The thermodynamic boundary of photocatalytic reduction reactions is determined by the energy of the excited electrons from the conduction band, specifically the energetic position of the conduction band edge at the semiconductor‐electrolyte interface.^[^ ^13^, ^14^ ^]^ For example, the conduction band position of anatase bulk TiO_2_ allows for reactions such as 2H^+^ + 2e^−^ → H_2_ or the formation of O_2_^−*^ from O_2_, as the conduction band edge is approximately 0.1–0.2 eV negative to the corresponding electrochemical reduction potentials. A possible strategy to shift the band energy level of a semiconductor and thus expand the thermodynamic limit of the photocatalytic reactions involves leveraging quantum confinement (QC) effects that lead to a widening of the band gap.^[^ ^15^, ^16^, ^17^, ^18^ ^]^ This effect is widely used in quantum well structures (such as GaAs/AlGaAs) or quantum dots of CdSe, InP, or PbS to tune optical properties. For TiO_2_, however, QC effects have been seldom reported, likely due to the high effective mass of charge carriers in titania.^[^ ^19^, ^20^ ^]^ Accordingly, for 2D TiO_2_, quantum confinement occurs only for extremely thin layers, and some doubts exist on the practical usefulness of QC effects for TiO_2_‐based devices.^[^ ^19^ ^]^ Another critical aspect of TiO_2_ is that, in virtually all forms of synthesis, an oxygen‐deficient material and therefore an n‐type material is obtained which typically has a relatively low mobility for hole carriers; this presents a significant challenge for achieving high photocatalytic performance.^[^ ^21^, ^22^, ^23^, ^24^ ^]^

Except for intrinsic features, additionally, for many photocatalytic reduction reactions, the placement of cocatalysts on the semiconductor surface is necessary to achieve a reasonable electron transfer kinetics; often this cocatalyst is a noble metal, such as Pt, which can facilitate charge carrier transfer and critical steps in the surface reaction.^[^ ^24^, ^25^, ^26^, ^27^, ^28^ ^]^ Most recently, noble metals in the form of single atoms (SAs) have emerged as a cocatalyst, not only because of a maximum in atom‐utilization efficiency but also as often the selectivity of reactions can be altered to desired products.^[^ ^29^, ^30^, ^31^, ^32^, ^33^, ^34^ ^]^

In the present work, we address several of above issues of traditional TiO_2_ photocatalysts by introducing p‐type TiO_2_ nanotubes (p‐TiO_2_ NTs) that show adjustable QC effects and we use these effects to enable a photocatalytic reaction previously deemed inaccessible using TiO_2_, that is photocatalytic ammonia production via nitrate reduction^[^ ^35^, ^36^ ^]^ (Figure 1a). Additionally, loading this QC‐substrate with Pt SAs not only provides a significant efficiency increase but also a remarkable shift in reaction selectivity.

**Figure 1:**
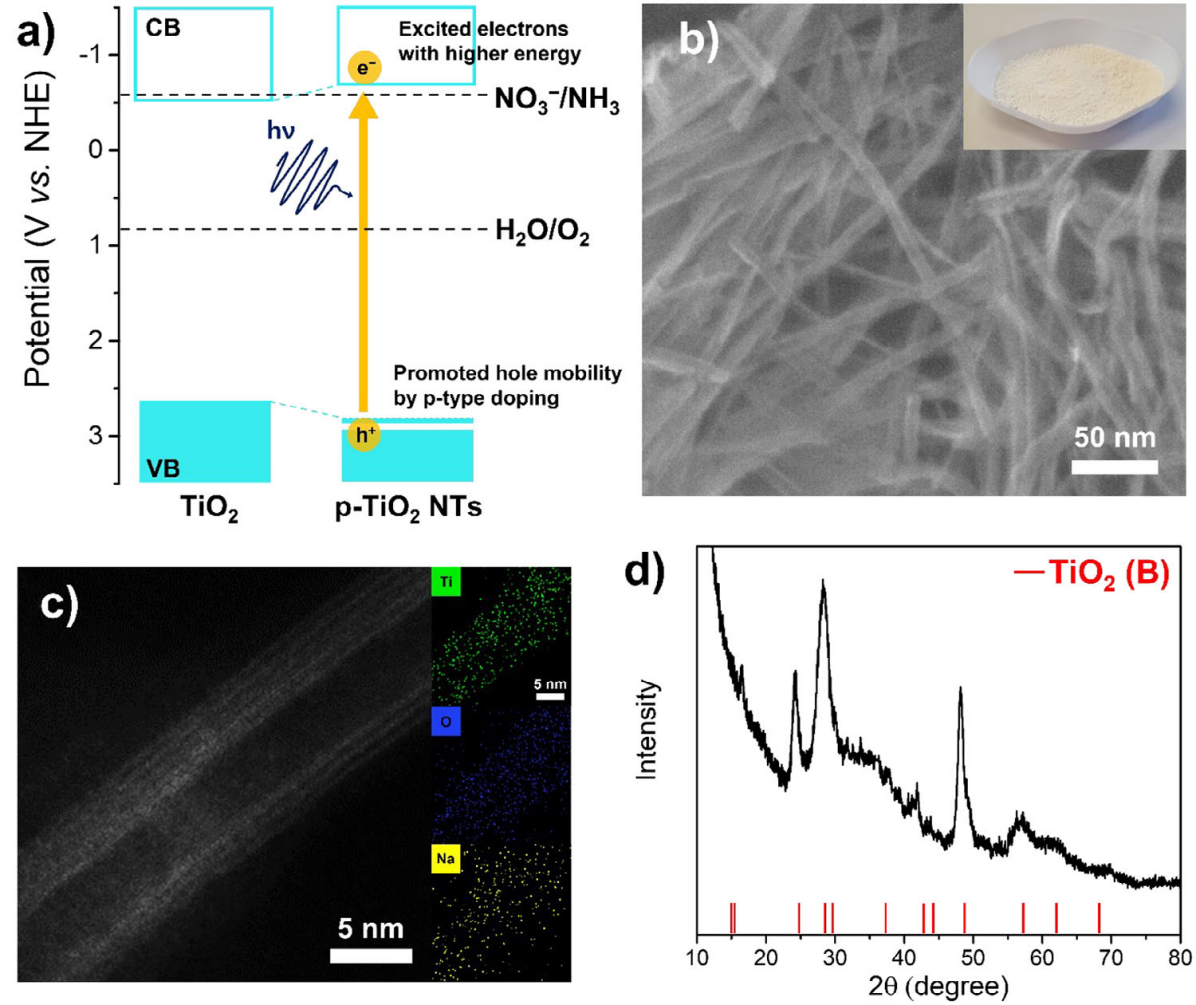
a) Schematic illustrations of photocatalytic nitrate reduction to ammonia at pH 7. The position of the conduction band and valence band of p‐TiO_2_ NTs is shown based on the band gap estimation and Mott–Schottky analyses. b) SEM (Inset: optical image of p‐TiO_2_ NTs powder), c) HAADF‐STEM, EDS mapping images, and d) XRD pattern of p‐TiO_2_ NTs. The positions of the reference were taken from the ICDD database.

The concept demonstrated here is not only of high practical significance for nitrate reduction pathways towards NH_3_ production but it also demonstrates the feasibility of tuning thermodynamics of titania‐based photocatalysis, and hence the reaction spectrum and selectivity of TiO_2_‐based photocatalytic reactions, and thus opens new directions for this classic photocatalyst.

In a first step, we synthesized TiO_2_ NTs through a hydrothermal approach by a modified process reported in literature.^[^ ^37^ ^]^ Synthesis details are described in the Experimental Section. The reaction product is a white powder (inset of Figure 1b) that consists of nanotubes with a length of several micrometers and an outer diameter of approximately 10 nm, as apparent from the scanning electron microscopy (SEM) images in Figure 1b. High‐angle annular dark‐field‐scanning transmission electron microscopy (HAADF‐STEM) image (Figure 1c) shows the individual tubes to consist of a layered (or rolled) sheet structure – well in line with literature^[^ ^37^ ^]^ – with a wall thickness of 2.7 nm. Energy‐dispersive X‐ray spectroscopy (EDS) mapping images (Figure 1c) confirm an elemental composition consistent with TiO_2_. Noteworthy is the presence of uniformly distributed Na throughout the entire nanotubes at a concentration of 4.46 at%. The presence of sodium was further confirmed by X‐ray photoelectron spectroscopy (XPS), as shown in Figure S1, with Na concentration of 5.14 at% in the nanotube sample. X‐ray diffraction (XRD) of the nanotubes (Figure 1d) shows a pattern that is well in line with the monoclinic structure of TiO_2_ (B). The peaks at 15.1°, 24.9°, 28.5°, 29.7°, 37.4°, 43.5°, 44.3°, 48.1°, 57.2°, 62.9°, and 68.0° can be assigned to (001), (110), (002), ($\bar{4}$01), (401), (003), (610), (020), (022), (11$\bar{4}$), and (023) diffraction planes of the TiO_2_ (B) phase (ICDD 046‐1237).

Figure 2a shows incident‐photon‐to‐current efficiency (IPCE) measurements for an electrode fabricated from the nanotube powder and the corresponding Tauc plot for an indirect electron transition yields a band gap of approximately 3.5 eV, which is significantly larger than that of TiO_2_ bulk materials (3.0–3.2 eV). The band gap of the nanotubes was additionally confirmed to be ≈ 3.5 eV by diffuse reflectance spectroscopy (Figure S2). It should be noted that a widened band gap for 3D TiO_2_ nanomaterials is rarely reported.^[^ ^19^, ^20^ ^]^ In general, QC effects and resulting band gap widening occur for TiO_2_ only for extremely thin layers and thin‐walled nanotubes of a lower dimensionality.^[^ ^19^, ^38^ ^]^ Remarkably, the photoelectrochemical experiments further show that these tubes have a p‐type electrical characteristic. Unlike other conventional titania and TiO_2_ (B) materials, p‐TiO_2_ NTs exhibit transient cathodic photocurrents as shown in Figure 2b. In addition, photocurrent‐voltage plots in Figure 2c show the typical behavior of a p‐type semiconductor with cathodic photocurrents that increase progressively with an increasing negative potential.^[^ ^21^, ^39^ ^]^ The p‐type conductivity can be further verified by Mott–Schottky analyses, which show linear plots with a negative slope (Figure 2d). At voltage anodic to 1.5 V (vs. Ag/AgCl), the TiO_2_ materials get deteriorated. The corresponding flat‐band potential (*U*_fb_) results as *U*_fb_ = 2.8 V versus NHE at pH 7 and a carrier density (*N*_d_) is listed in Table S1 (The energetic position of conduction band of p‐TiO_2_ NTs is estimated to be −0.7 V versus NHE at pH 7 based on the band gap and flat‐band potential evaluation). This means the band gap is widened in the cathodic as well as anodic direction. The p‐type conductivity of the p‐TiO_2_ NTs can be assigned to the incorporation of Na because group IA elements, especially Li and Na, can provide shallow acceptor levels close to the valence band maxima.^[^ ^40^, ^41^ ^]^ To estimate the specific surface area of p‐TiO_2_ NTs, Brunauer–Emmett–Teller (BET) analyses were conducted (Figure S3). The p‐TiO_2_ NTs exhibited a typical type IV isotherm, which might be attributed to the lumen of the nanotubes, and the corresponding surface area was determined to be 74.3 m^2^ g^−1^.

**Figure 2:**
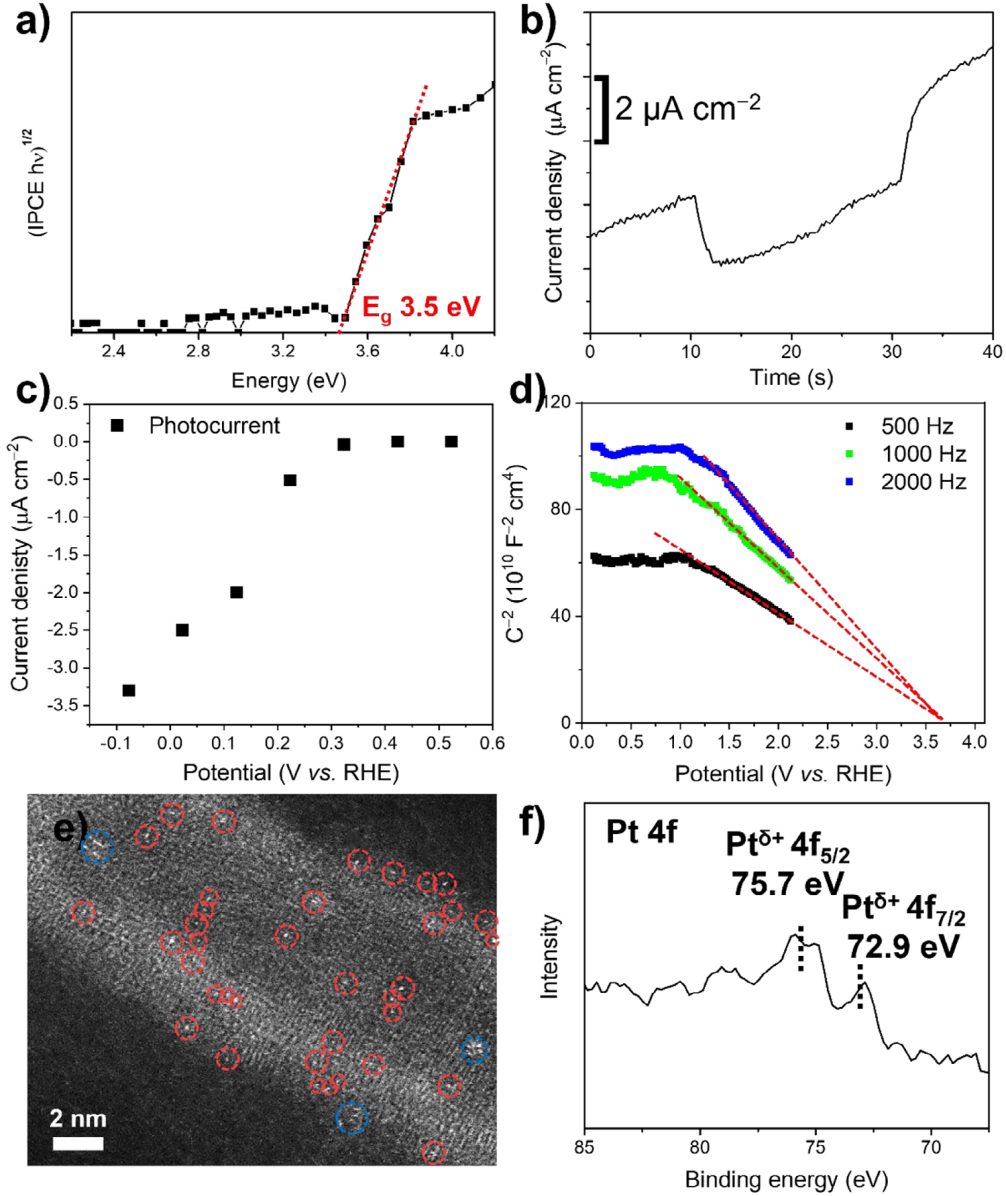
a) Band gap estimation by IPCE measurements and b) transient photocurrent curve at −0.5 V versus Ag/AgCl (0.1234 V vs. RHE) under 320 nm monochromatic irradiation for p‐TiO_2_ NTs. c) Photocurrents of p‐TiO_2_ NTs at various potentials under 320 nm monochromatic irradiation. d) Mott–Schottky plots for p‐TiO_2_ NTs. e) HAADF‐STEM image with highlighted SAs (red circles) and rafts (blue circles), and f) Pt 4f XPS spectrum of Pt SAs/p‐TiO_2_ NTs synthesized with 0.05 mM H_2_PtCl_6_ solution.

These TiO_2_ NTs were then decorated with Pt SAs by a reactive deposition method^[^ ^31^, ^32^, ^33^, ^34^ ^]^ using a 0.05 mM H_2_PtCl_6_ solution, creating Pt SAs. (The synthesis procedure of these Pt SAs/p‐TiO_2_ NTs is described in Experimental Section in detail). As expected, SEM images of the Pt SAs/p‐TiO_2_ NTs (Figure S4) do not show any noticeable change in morphology, i.e., no formation of Pt nanoparticles can be observed after the deposition. Nevertheless, HAADF‐STEM images confirm the successful decoration with well separated Pt SAs and some few‐atom rafts on the p‐TiO_2_ NTs substrates (Figure 2e). An evaluation of the HAADF‐STEM images shows that Pt SAs were decorated at a density of approximately 4.2 x 10^5^ µm^−2^.

To identify the chemical state of the deposited Pt species, XPS of Pt SAs/p‐TiO_2_ NTs was performed for the Pt 4f region (Figure 2f). The Pt SAs/p‐TiO_2_ NTs displays a doublet peak of Pt 4f_7/2_ and 4f_5/2_ at 72.9 and 75.7 eV, respectively, indicating the presence of two‐fold oxygen‐coordinated Pt SAs with a formal charge (δ+) around δ ≈ 2.^[^ ^31^, ^32^, ^33^, ^34^, ^42^ ^]^

To investigate the feasibility of the concept shown in Figure 1a, i.e., to use the widened gap (3.5 eV) and elevated conduction band position (−0.7 V vs. NHE at pH 7) of the p‐TiO_2_ NTs, we selected a technologically important reaction that normally cannot be carried out with TiO_2_‐based photocatalysts, the photocatalytic ammonia production from nitrate.^[^ ^35^, ^36^ ^]^ This reduction reaction (NO_3_^−^ + 9H^+^ + 8e^−^ → NH_3_ + 3H_2_O, −0.58 V vs. NHE at pH 7) was evaluated under 275 nm LED irradiation (10 mW cm^−2^) and compared with the classic benchmark P25 Degussa catalyst (Pt SAs/P25) with a typical n‐type conductivity and a classic band gap of 3.0–3.2 eV.^[^ ^11^, ^43^ ^]^ To explore the tunability of the QC effect, we also fabricated hydrothermal NTs with slightly thicker walls of around 3.7 nm (p‐TiO_2_ thick NTs) and these nanotubes showed a band gap of approximately 3.4 eV (Figure S5). It is noteworthy that the experimental band gap data of the nanotubes are well‐matched with the estimation by Brus effective mass approximation (Figure S6). Details of the estimation by Brus model for quasi‐1D nanotubes are described in the Supporting Information. Figure 3a shows the amounts of ammonia produced from various catalysts over 8 h, and corresponding ammonia production rates are summarized in Figure 3b. Evidently, the Pt SAs/p‐TiO_2_ NTs outperforms the Pt SAs/P25 and Pt SAs/p‐TiO_2_ thick NTs in photocatalytic activity. Interestingly, P25 with Pt SAs decoration also exhibited slight ammonia production even though photocatalytic nitrate reduction to ammonia is thermodynamically impossible with the conduction band level of this form of TiO_2_. However, the noble metal cocatalysts may enable minor amount of nitrate reduction by facilitating the hydrogenation with chemisorbed hydrogen on the noble metal surface.^[^ ^44^, ^45^ ^]^ Indeed, even with 24 h irradiation of the LED, no ammonia was detectable for the photocatalytic nitrate reduction for bare P25, which is well in line with other reported literature.^[^ ^45^, ^46^, ^47^ ^]^ On the contrary, Pt‐free p‐TiO_2_ NTs shows clearly detectable ammonia generation (15.5 µmol g^−1^) under 24 h LED illumination., i.e., the widened band gap is sufficient to alter the thermodynamic energy (reductive power) of the photogenerated charge carriers. In addition, the durability and recyclability of the Pt SAs/p‐TiO_2_ NTs for photocatalytic nitrate reduction were examined. After the photocatalysis, the tubular morphology of TiO_2_ was well‐maintained while there were some agglomerations and reduction from Pt SAs to metallic nanoparticles (Figure S7a,b), which is consistent with previous literature of SA photocatalysts.^[^ ^34^ ^]^ However, the Pt SAs/p‐TiO_2_ NTs displayed excellent recyclability for repeated photocatalytic cycles (Figure S7c), suggesting the remaining Pt SAs play a dominant role in the photocatalytic performance.^[^ ^34^ ^]^

**Figure 3:**
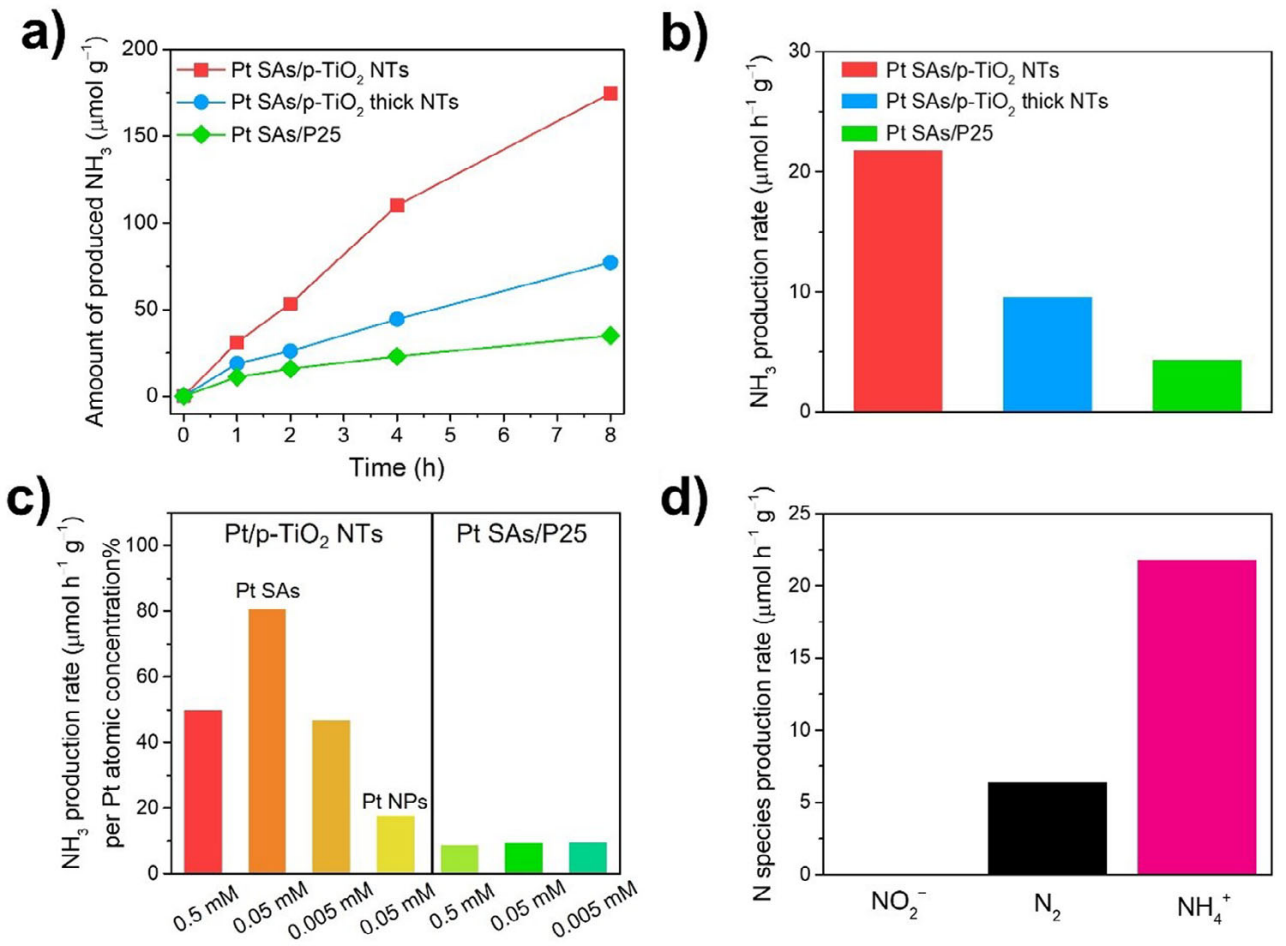
a) Time‐course ammonia production and b) corresponding photocatalytic ammonia production rate of Pt SAs/p‐TiO_2_ NTs, Pt SAs/p‐TiO_2_ thick NTs, and Pt SAs/P25 (0.05 mM) under 275 nm LED irradiation. c) Pt‐normalized ammonia production rate from nitrate reduction over various catalysts. d) Resultant nitrogen species after photocatalytic nitrate reduction of Pt SAs/p‐TiO_2_ NTs (0.05 mM).

Furthermore, to elucidate the role of Na and the resulting p‐type conductivity in the photocatalytic reaction of the Pt SAs/p‐TiO_2_ NTs, n‐type TiO_2_ nanotubes (n‐TiO_2_ NTs) with similar morphology (Figure S8a), but minor amounts of Na species (Figure S1) were prepared by a modified post‐treatment process.^[^ ^48^ ^]^ The n‐type conductivity was confirmed by the positive photocurrent and Mott–Schottky plots (Figure S8b,c), indicating the p‐type conductivity originates from the Na dopants. The according *U*_fb_ and *N*_d_ by Mott–Schottky analyses are summarized in Table S1. Then, Pt SAs were deposited on the n‐TiO_2_ NTs, namely Pt SAs/n‐TiO_2_ NTs, and their photocatalytic activity for ammonia production was evaluated. Apparently, the performance is inferior to that of Pt SAs/p‐TiO_2_ NTs (Figure S8d), suggesting the advantageous role of the Na species and p‐type conductivity in our catalytic system.

To investigate the influence of the Pt SA loading concentration in photocatalysis, ammonia production rates of Pt SAs‐decorated p‐TiO_2_ NTs and P25 with different concentrations of the Pt precursor (0.5, 0.05, and 0.005 mM) were evaluated (Figure 3c and S9). The surface density of SAs in p‐TiO_2_ NTs for these conditions is estimated to be 1.2 x 10^6^, 4.2 x 10^5^, and 2.3 x 10^5^ µm^−2^, respectively, based on the HAADF‐STEM images and XPS analyses (Table S2). Although Pt SAs/p‐TiO_2_ NTs with 0.5 mM H_2_PtCl_6_ solution displayed the highest production rate, which may be attributed to significantly higher Pt loading, it is apparent that Pt SAs/p‐TiO_2_ NTs synthesized with 0.05 mM H_2_PtCl_6_ solution exhibited the most outstanding photocatalytic performance if the activity is normalized to the Pt content based on the Pt atomic concentrations from XPS data (Table S2). If Pt nanoparticles, instead of Pt SAs, are decorated on the p‐TiO_2_ NTs (Pt NPs/p‐TiO_2_ NTs, Figure S10) by photodeposition (see Supporting Information for details), an inferior photocatalytic performance to Pt SAs/p‐TiO_2_ NTs is observed, in terms of absolute activity but even more so when the activity is normalized to Pt loading.

Moreover, it is important to explore other nitrogen species that can be generated during the multiple reduction reactions of nitrate, such as nitrite (NO_2_^−^) and nitrogen gas (N_2_), which are the major reaction side products in the nitrate reduction.^[^ ^47^, ^49^, ^50^, ^51^ ^]^ In the photocatalysis of Pt SAs/p‐TiO_2_ NTs, Pt SAs/n‐TiO_2_ NTs, Pt SAs/P25, and Pt NPs/p‐TiO_2_ NTs (Figure 3d and S11), nitrite was not detected for Pt SAs/p‐TiO_2_ NTs, Pt SAs/n‐TiO_2_ NTs, and Pt SAs/P25, while nitrogen gas was produced from the catalysts with relatively lower amount compared to ammonia for Pt SAs/p‐TiO_2_ NTs. On the other hand, Pt NPs/p‐TiO_2_ NTs exhibited the generation of all three nitrogen species, favoring nitrite rather than ammonia. This clearly shows that the use of Pt SAs instead of NPs significantly shifts the selectivity of the reaction toward the desired NH_3_ product. The estimated selectivity to ammonia based on the nitrogen mole balance for Pt SAs/p‐TiO_2_ NTs, Pt SAs/n‐TiO_2_ NTs, Pt SAs/P25, and Pt NPs/p‐TiO_2_ NTs is 62.96, 49.17, 43.57, and 17.82%, respectively. In addition, it is worth mentioning that our Pt SAs‐decorated TiO_2_ photocatalysts are expected to be advantageous in selectively capturing the generated ammonia since they do not generate toxic nitrite and only produce N_2_ as side product, which is sparingly soluble in water.

To further investigate the role of Pt SAs and p‐TiO_2_ NTs in the photocatalytic reaction of Pt SAs/p‐TiO_2_ NTs, other nitrogen species were examined for bare p‐TiO_2_ NTs and P25 without Pt SA cocatalysts under 24 h LED irradiation. As in the case of the ammonia production, P25 did not show any nitrogen species formation, which is well in line with other literature.^[^ ^46^, ^47^ ^]^ However, p‐TiO_2_ NTs exhibited the photocatalytic nitrate reduction mostly to nitrite (Figure S11d, selectivity: 76.47%). This suggests Pt SAs not only accelerate the overall nitrate reduction process, but have a major role in expediting the subsequent reduction and hydrogenation from nitrite to ammonia. Given the significant enhancement of photocatalytic activity and selectivity by Pt SAs decoration and strong adsorption of N, O, and H atoms on Pt, it is expected that the redox reaction would favorably occur on Pt SA sites and surrounding Ti atoms.^[^ ^44^, ^52^, ^53^ ^]^ In addition, since bare p‐TiO_2_ NTs are able to reduce nitrate mostly to nitrite, it is also possible to have two separate reduction steps (i.e., initial nitrate to nitrite reduction on TiO_2_ surface and further reduction on Pt SAs) in Pt SAs/p‐TiO_2_ NTs. Nonetheless, it is clear that regardless of the pathway, Pt SAs are crucial as active sites in the photocatalytic reactions.

It is also important to note that all reactions in our photocatalysis experiments were conducted without any hole scavengers. Most studies with TiO_2_ materials (with or without cocatalysts) use hole scavengers (e.g., methanol, humic acid, formic acid, and etc.) for photocatalytic nitrate reduction because the water oxidation reaction is kinetically sluggish. Moreover, an aqueous CO_2_ radical anion (i.e., CO_2_^−^), typically generated during the oxidation of these hole scavengers, is formed in such reactions that enables nitrate reduction which otherwise is not thermodynamically possible with an unmodified conduction band level of TiO_2_.^[^ ^47^, ^49^, ^50^, ^51^ ^]^ In this regard, we describe in the present work that Pt SAs/p‐TiO_2_ NTs can serve as an ideal platform for ammonia production in plain nitrate solution by addressing these issues. As illustrated in Figure 1a, the Pt SAs/p‐TiO_2_ NTs possess more pronounced hole mobility originated from their p‐type conductivity, which can facilitate the water oxidation and overall redox reactions, in contrast to Pt SAs/n‐TiO_2_ NTs and Pt SAs/P25 exhibiting n‐type conductivity. Additionally, excited electrons in the Pt SAs/p‐TiO_2_ NTs are thermodynamically confined so that they can enable the nitrate reduction reaction to ammonia with their relatively higher energy and enhanced interfacial charge transfer compared to the Pt SAs/P25. We believe, in the present work we show unique, highly relevant photocatalytic features that can be established by tuning the most classic titania photocatalyst in conduction type, band‐edge positions, and the use of single atom cocatalysts.

In conclusion, the present work shows that the titania based photocatalyst (p‐TiO_2_ NTs decorated with Pt SAs) allows for highly selective photocatalytic ammonia production through nitrate reduction reaction without any hole scavengers. The unique electronic properties and structures of p‐TiO_2_ NTs (p‐type conductivity and QC) enable a remarkable photocatalytic activity toward ammonia compared to those of the benchmark catalysts, Pt SAs/P25. We show that indeed the QC effect and the band gap of the nanotubes can be controlled by adjusting the thickness of the titania nanotube. Furthermore, we show that the use of Pt SAs can drastically enhance the selectivity toward the desired products. In summary, the present work demonstrates that unique, highly relevant photocatalytic features can be established by tuning the most classic titania photocatalyst in conduction type, band‐edge positions, and the use of single atom cocatalysts.

## Conflict of Interests

The authors declare no conflict of interest.

## Supporting information



Supporting Information

## Data Availability

The data that support the findings of this study are available in the supplementary material of this article.
