# Supplementary material for: p‐Type TiO2 Nanotubes: Quantum Confinement and Pt Single Atom Decoration Enable High Selectivity Photocatalytic Nitrate Reduction to Ammonia
Source: Angew Chem Int Ed Engl. 2025 Mar 30;64(22):e202415865. doi: 10.1002/anie.202415865 (PMC12105680; doi:10.1002/anie.202415865)
Supplement: Supplementary file 1 — Supporting Information [file ANIE-64-e202415865-s001.pdf]

Supporting Information

p-Type TiO_2 Nanotubes: Quantum Confinement and Pt Single Atom Decoration Enable High Selectivity Photocatalytic Nitrate Reduction to Ammonia

Hayoon Jung, [a, b] Hyesung Kim, [a] Johannes Will, [c] Erdmann Spiecker, [c] and Patrik Schmuki [a, b] *

[a] Department of Materials Science and Engineering, WW4-LKO, Friedrich-Alexander-Universität Erlangen-Nürnberg, Martensstraße 7, 91058 Erlangen, Germany

[b] Regional Centre of Advanced Technologies and Materials, Czech Advanced Technology and Research Institute (CATRIN), Palacký University, Šlechtitelů 27, 78371 Olomouc, Czech Republic

[c] Institute of Micro- and Nanostructure Research & Center for Nanoanalysis and Electron Microscopy (CENEM), IZNF, Friedrich-Alexander-Universität Erlangen-Nürnberg, Cauerstraße 3, 91058 Erlangen, Germany

E-mail: schmuki@ww.uni-erlangen.de

Experimental Section

Chemicals and Materials

TiO_2 anatase nanopowder (Sigma–Aldrich, 99.7%), NaOH (Carl Roth, ≥99%), HCl (Carl Roth, 37%), P25 (Sigma–Aldrich, ≥99.5%), $H_2PtCl_6{\cdot}6H_2O$ (Metakem, 40.17% metallic Pt weight concentration), Na_2SO_4 (Carl Roth, ≥99%), KNO_3 (Sigma–Aldrich, ≥99.0%), acetonitrile (Carl Roth, ≥99.8%) were used as received without any purification. Aqueous solutions were prepared using ultrapure water with a resistivity exceeding 18.2 MΩ·cm.

Synthesis of p-TiO_2 NTs

p-TiO_2 NTs were prepared by a hydrothermal method. First, 3.0 g of TiO_2 anatase nanopowder was dispersed in NaOH aqueous solution (10 M, 40 mL). The reaction solution was then transferred into 100 mL Teflon autoclave and heated at 150 °C for 48 h. The resultant powder was filtered with deionized water and HCl aqueous solution (0.1 M), and dried at 70 °C for further usage. For synthesizing thick p-TiO_2 NTs, a similar method was employed, however with 4.5 g of TiO_2 anatase nanopowder and heating for 72 h.

Synthesis of n-TiO_2 NTs

n-TiO_2 NTs were prepared by an identical hydrothermal method for p-TiO_2 NTs, but with different post-treatment after the heating.[1] The resultant powder was immersed in 1 M of HCl aqueous solution for 2 h, then rinsed with deionized water, and dried at 70 °C for further usage.

Pt SAs and NPs deposition

To deposit Pt SAs on TiO_2 samples (p-TiO_2 NTs, P25, and n-TiO_2 NTs), 40 mg of TiO_2 powder was dispersed in 200 mL water. Then, the solution was purged with argon for 15 min and H_2PtCl_6 was added to the solution to make the overall Pt precursor concentration of 0.005, 0.05, and 0.5 mM. The reaction solution was stirred for 1 h in dark and the resultant samples were washed by centrifugation at 4,000 rpm for 20 min. For photodeposition of Pt NPs, 20 mg of the TiO_2 powder was dispersed in 20 mL of methanol aqueous solution (50 vol%) and the resultant solution was purged with argon for 15 min. Then, H_2PtCl_6 was added to the solution to make the overall Pt precursor concentration of 0.05 mM. UV irradiation (λ = 275 nm, power density = 10 mW cm^{-2}) was conducted for 24 h and the samples were washed by centrifugation at 4,000 rpm for 20 min.

Characterization

A Hitachi S-4800 scanning electron microscope was used to obtain SEM images. HAADF-STEM and EDS elemental mapping images were collected by a high-resolution transmission electron microscope (Spectra 200 C-FEG, Thermo Fisher Scientific) with a probe-correction and a cold field emission gun (X-CFEG). The XRD pattern of p-TiO_2 NTs was recorded by a X-ray diffractometer (X‘pert Philips MPD with a Panalytical X‘celerator detector) with graphite monochromatized Cu Kα radiation (λ = 0.15406 nm). XPS measurements were carried out by X-ray photoelectron spectrometer (PHI 5600, US) with monochromatic Al Kα radiation (1486.6 eV, 300 W) to analyze the chemical composition of the samples. Diffuse reflectance

spectroscopy was conducted by Avantes AvaSpec-2048L spectrometer equipped with Avantes AvaLight-DH-S-BAL light source.

Brunauer-Emmett-Teller (BET) surface area estimation

The BET theory was employed to assess the specific surface area (S_{BET}) of p-TiO_2 NTs. N_2 adsorption/desorption isotherms were recorded at 77 K on a volumetric gas adsorption analyzer (Autosorb iQ XR, Anton-Paar Quanta Tec, USA) up to 0.965, following Rouquerol criteria. High purity (99.999%) N_2 and He gases were utilized during the measurements. Prior to analysis, the sample underwent degassing under high vacuum (10^{-7} Pa) for 12 h.

Photoelectrochemical analysis

(Photo)electrochemical analyses, including band gap estimation with IPCE, transient photocurrent response, and Mott-Schottky analyses, were conducted using a 3-electrode cell configuration. The cell comprised the nanotubes sample as a working electrode, Pt plate as a counter electrode, and Ag/AgCl as a reference electrode. In case of photoelectrochemical experiments, a 0.1 M Na_2SO_4 aqueous solution (Ar-purged) served as the electrolyte. the irradiated and electrolyte-exposed area for each sample was 0.5 cm^2. IPCE data were collected under a constant potential of −0.5 V (*vs.* Ag/AgCl) across the illumination wavelength range of 250–600 nm. The measurements utilized an Oriel 6365 150 W Xe-lamp equipped with an Oriel Cornerstone 7400 1/8 *m* monochromator as the light source. Transient photocurrent response was measured at constant potentials within the range from −0.1 to −0.7 V (*vs.* Ag/AgCl) at λ = 320 nm (power density = 0.15 mW cm^{-2}) using the same light source employed in the IPCE

measurements. In case of n-TiO_2 NTs, the photocurrent data were obtained under 0.5 V (*vs.* Ag/AgCl). Throughout the measurements, the samples underwent the following sequence: light off (10 s); light on (20 s); light off (10 s). In the Mott-Schottky analyses, capacitance was derived from the electrochemical impedance at each potential, which was measured by applying a potential perturbation with an amplitude of 10 mV and a frequency in the range of 1–10^4 Hz.

Photocatalysis

Photocatalytic nitrate reduction reaction was performed under UV irradiation (λ = 275 nm, power density = 10 mW cm^{-2}). 2.0 mg of catalysts were mixed in 10 mL of aqueous solution of KNO_3 (1 mM) in a quartz cell. The reaction solution was purged with Ar gas for 15 min to create anaerobic conditions. The amount of nitrogen species products was evaluated by ion chromatography (833 Basic IC plus and 930 Compact IC Flex, Metrohm). The amount of N_2 was estimated based on the mass (N) balance of the reactant and other products (NO_2^- and NH_4^+).

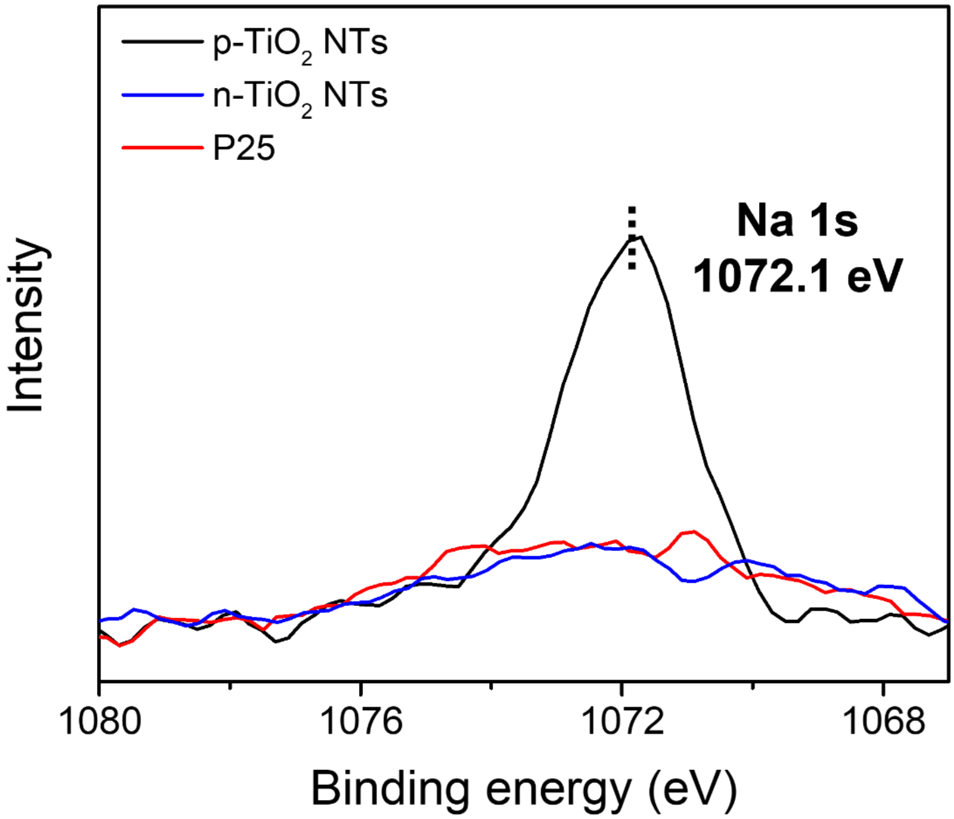

Figure S1. Na 1s XPS spectra of p-TiO_2 NTs, n-TiO_2 NTs, and P25.

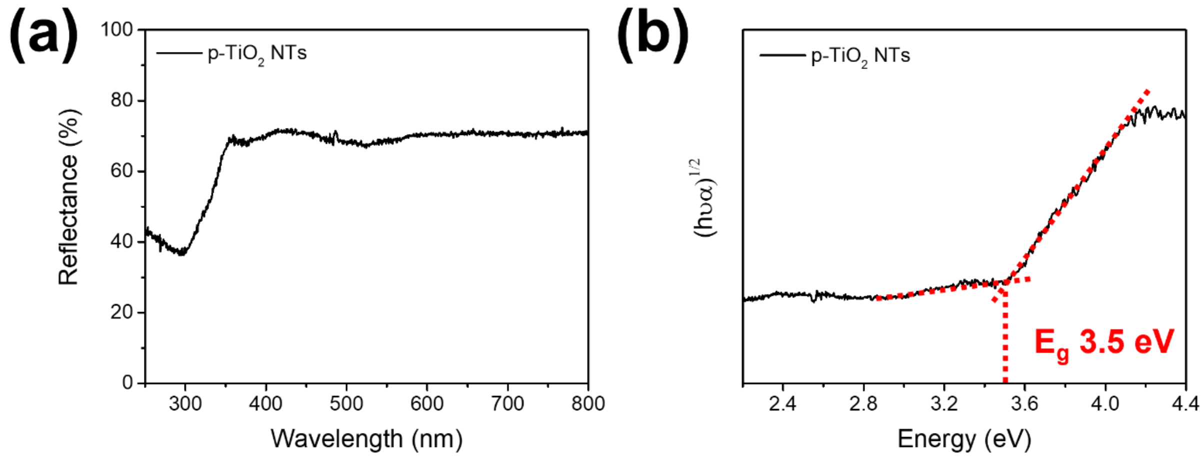

Figure S2. (a) Diffuse reflectance spectroscopy of p-TiO_2 NTs and (b) corresponding Tauc plot.

Brunauer–Emmett–Teller (BET) analysis

The surface area of p-TiO_2 NTs was determined using the BET theory with N_2 adsorption-desorption measurements. The N_2 adsorption-desorption isotherm plot (Figure S2a) exhibits a type IV isotherm with a hysteresis loop. The calculated specific surface area of the p-TiO_2 NTs, derived from the multi-point BET analysis, is approximately 74.315 m^2 g^{-1} (Figure S2b).

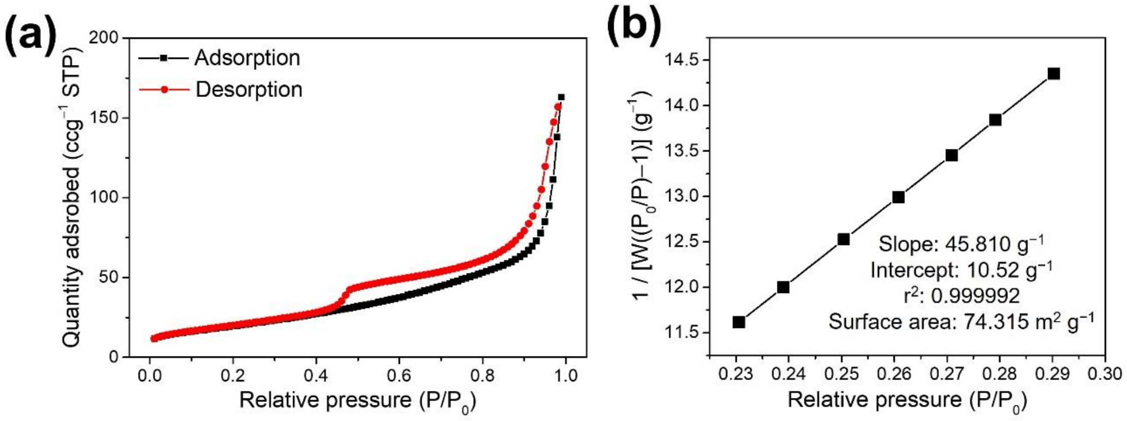

Figure S3. (a) Nitrogen adsorption/desorption isotherms and (b) multi-point BET plot for p-TiO_2 NTs.

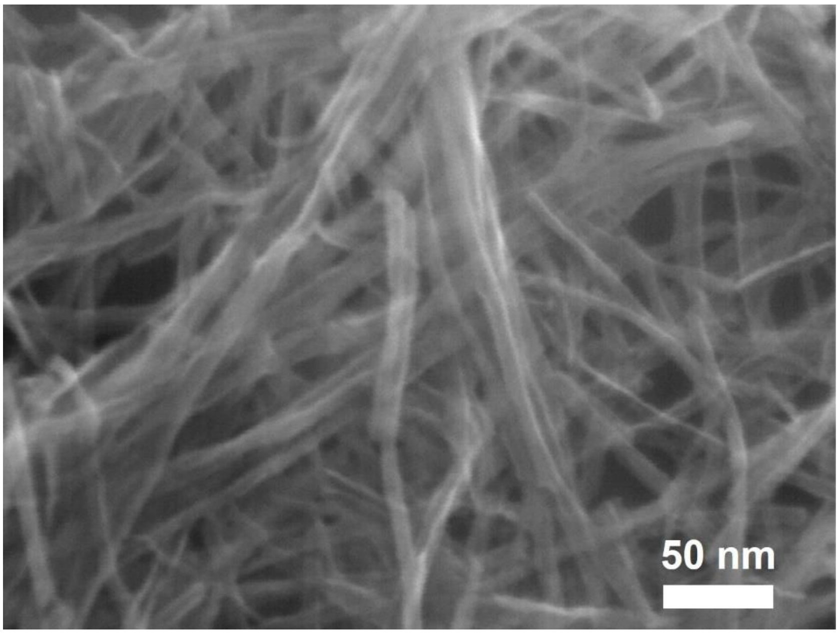

Figure S4. SEM image of Pt SAs/p-TiO_2 NTs.

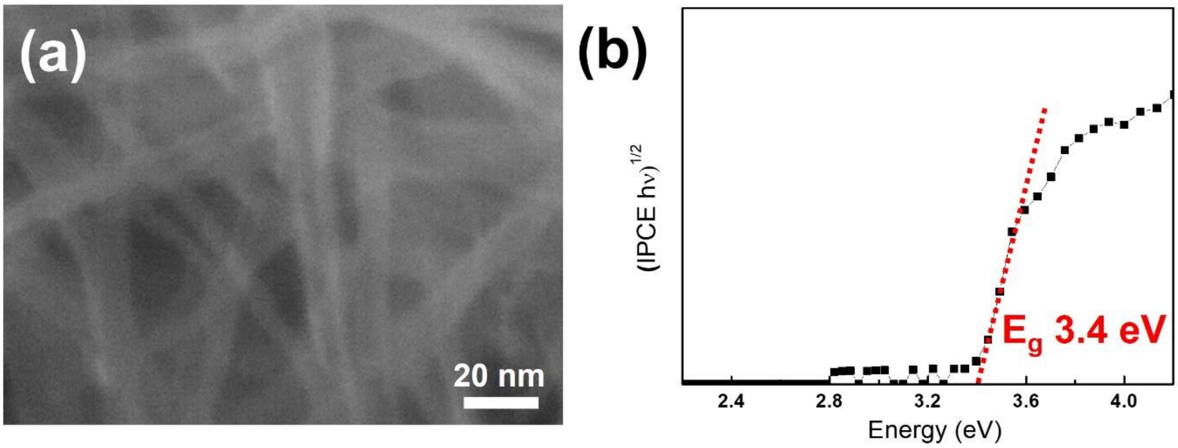

Figure S5. (a) SEM image and (b) band gap estimation by IPCE measurements of p-TiO_2 thick NTs.

Band gap shift estimation with Brus effective mass approximation

The band gap of quasi-1D TiO_2 nanotubes (E_g) with quantum confinement can be estimated by Brus effective mass approximation[2,3] as follows (equation 1):

$$E_g = E_{g,bulk} + \frac{h^2}{8m_0\mu}\left[\frac{1}{L^2} + \left(\frac{2}{d}\right)^2\right] \quad (1)$$

where $E_{g,bulk}$ is the band gap of bulk TiO_2, which is around 3.2 eV for TiO_2 (B),[4] h is plank constant, m_0 is the effective mass of electron, μ is the reduced effective mass of exciton, L is the wall thickness of the tubes (2.7 and 3.7 nm), and d is the diameter of the tubes (≈ 10 nm). The Brus model with μ of 0.20 was well-fitted with our experimental data.

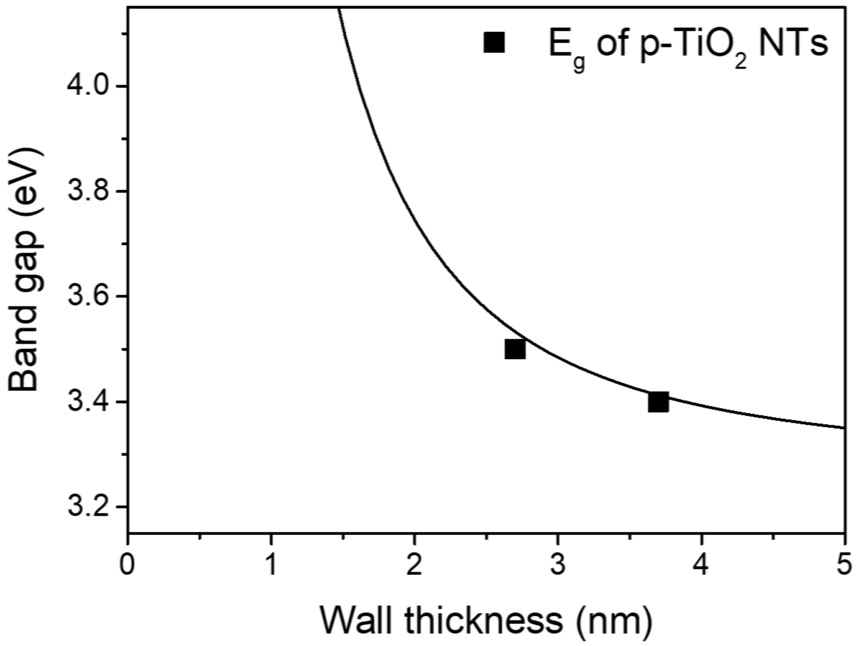

Figure S6. Band gap data of p-TiO_2 NTs and p-TiO_2 thick NTs, and expected band gap calculated by Brus effective mass approximation for quasi-1D nanotubes with reduced effective exciton mass of 0.20.

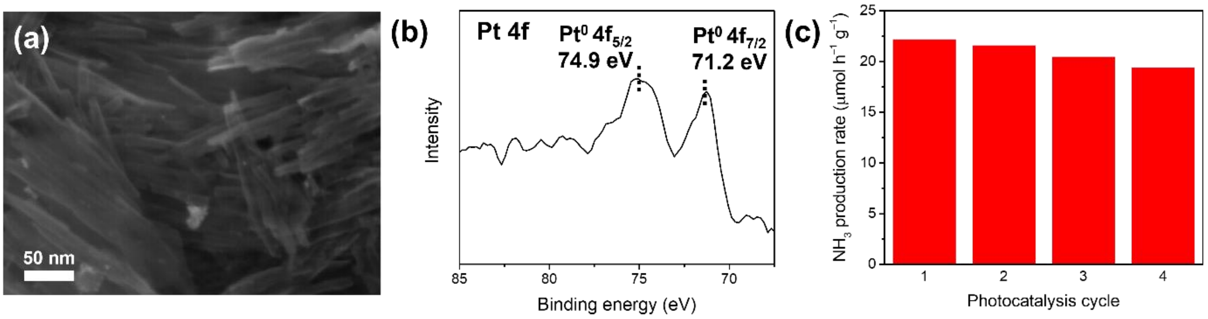

Figure S7. (a) SEM image and (b) Pt 4f XPS spectrum of Pt SAs/p-TiO_2 NTs after 8 h photocatalysis. (c) Recyclability test of Pt SAs/p-TiO_2 NTs for photocatalytic nitrate reduction. Each photocatalysis cycle was conducted for 8 h.

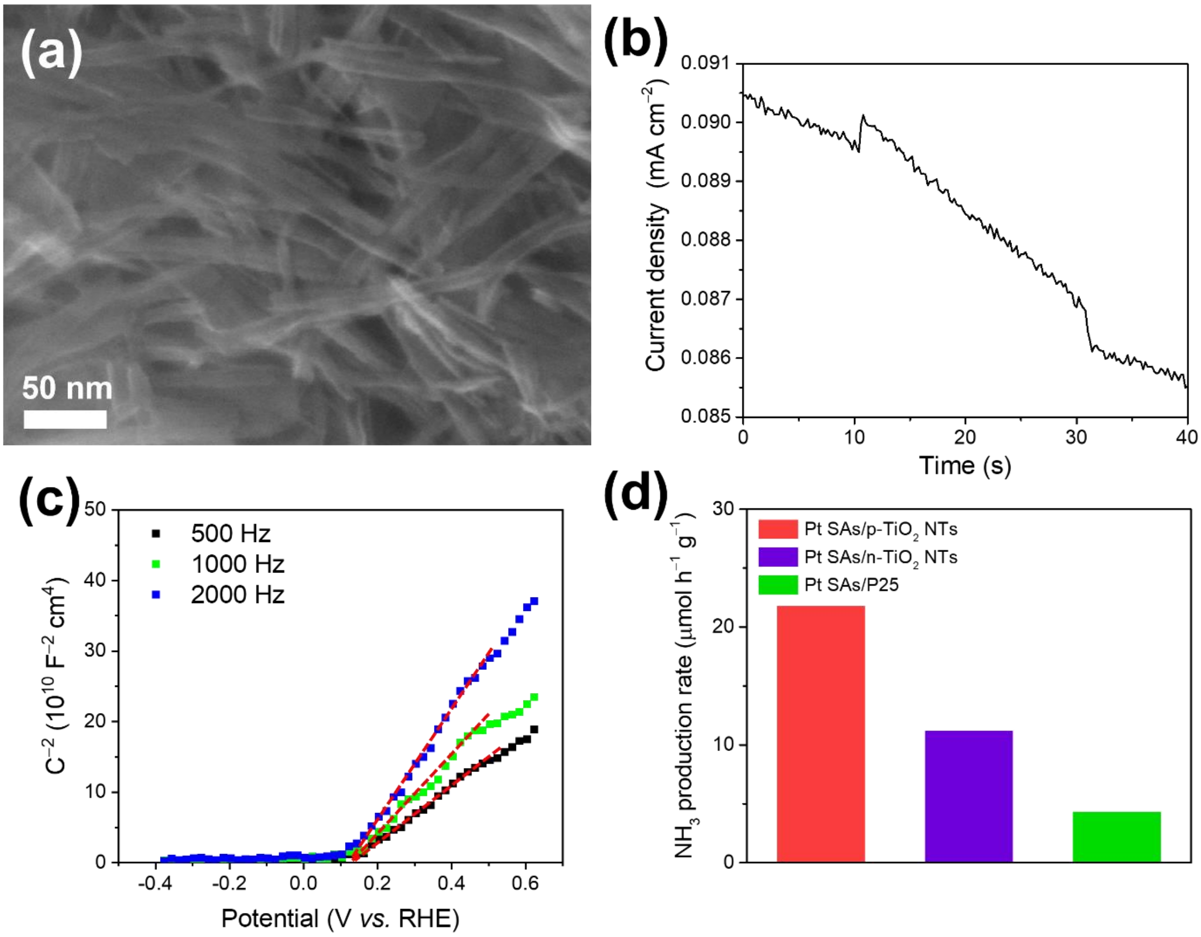

Figure S8. (a) SEM image, (b) transient photocurrent curve at 0.5 V *vs.* Ag/AgCl (0.1234 V *vs.* RHE) under 320 nm monochromatic irradiation, (c) Mott-Schottky plots of n-TiO_2 NTs. (d) Photocatalytic ammonia production rate of Pt SAs/n-TiO_2 NTs.

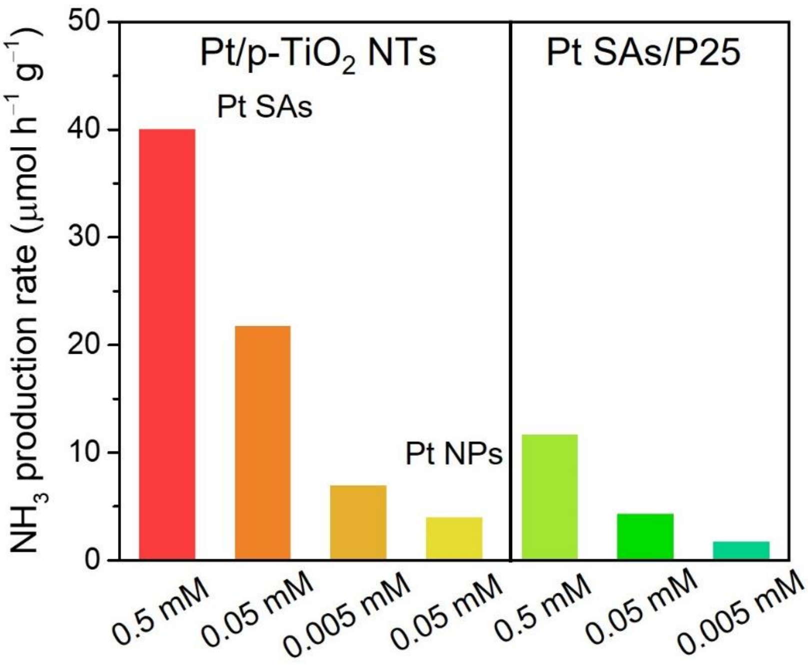

Figure S9. Photocatalytic ammonia production rate from nitrate reduction over various catalysts under 275 nm LED irradiation.

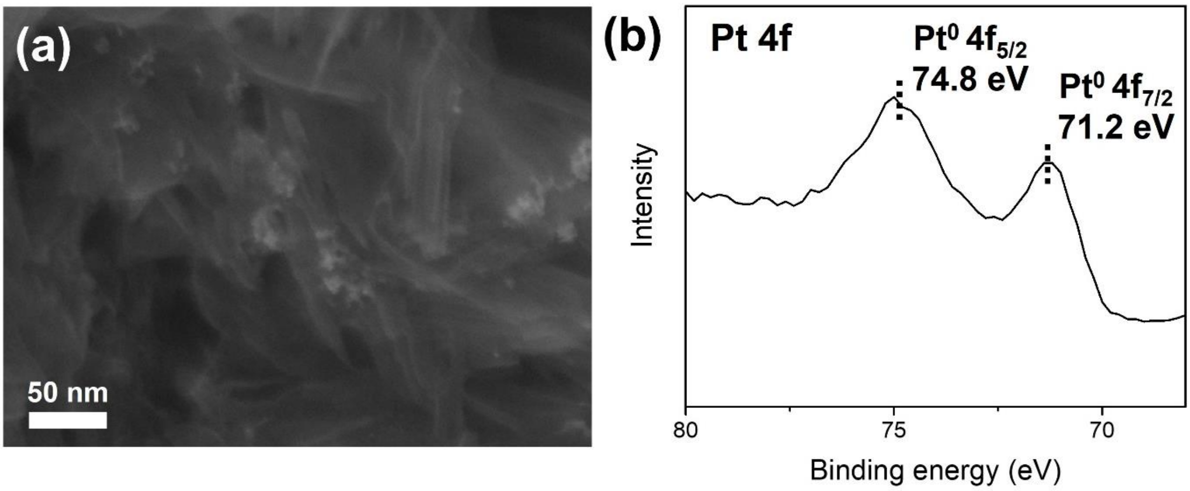

Figure S10. (a) SEM image and (B) Pt 4f XPS spectrum of Pt NPs/p-TiO_2 NTs.

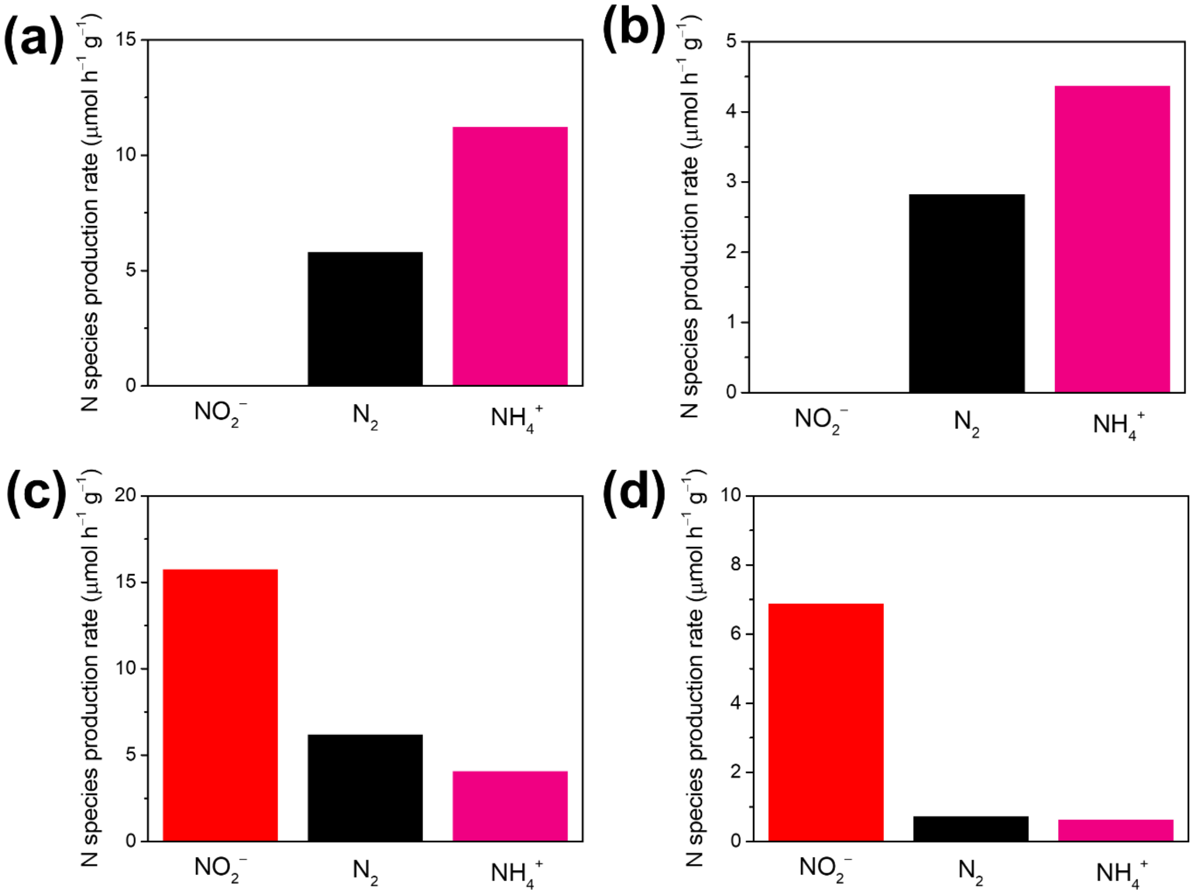

Figure S11. Nitrogen species production rate for (a) Pt SAs/n-TiO_2 NTs, (b) Pt SAs/P25, (c) Pt NPs/p-TiO_2 NTs, and (d) p-TiO_2 NTs under 275 nm LED illumination. For p-TiO_2 NTs, the LED was illuminated for 24 h, while for the others, for 8 h.

Table S1. Flat-band potential (U_{fb}) and carrier density (N_d) of p-TiO_2 NTs and n-TiO_2 NTs based on Mott-Schottky analyses.

Material	U_{fb} (V *vs.* NHE at pH 7)	N_d (cm^{-3})
p-TiO_2 NTs	2.8	6.08×10^{18}
n-TiO_2 NTs	−0.7	3.60×10^{18}

Table S2. Pt atomic concentrations of Pt SAs/p-TiO_2 NTs, Pt SAs/P25, and Pt NPs/p-TiO_2 NTs by XPS and estimated surface density of Pt SAs in Pt SAs/p-TiO_2 NTs.

Concentration of H_2PtCl_6 solution (mM)	Pt SAs/p-TiO_2 NTs		Pt SAs/P25 (at%)	Pt NPs/p-TiO_2 NTs (at%)
	(at%)	density (μm^{-2})		
0.5	0.80	1.2×10^6	1.33	
0.05	0.27	4.2×10^5	0.46	0.23
0.005	0.15	2.3×10^5	0.19	

References

[1] B. Poudel, W. Z. Wang, C. Dames, J. Y. Huang, S. Kunwar, D. Z. Wang, D. Banerjee, G. Chen, Z. F. Ren, *Nanotechnology* 2005, *16*, 1935-1940.

[2] L. E. Brus, *J. Chem. Phys.* **1983**, *79*, 5566-5571.

[3] D. V. Bavykin, F. C. Walsh, *Titanate and Titania Nanotubes: Synthesis, Properties and Applications,* Royal Society of Chemistry, **2009**.

[4] D. P. Kumar, N. L. Reddy, M. M. Kumari, B. Srinivas, V. D. Kumari, B. Sreedhar, V. Roddatis, O. Bondarchuk, M. Karthik, B. Neppolian, M. V. Shankar, *Sol. Energy Mater. Sol. Cells* **2015**, *136,* 157-166.
